# Factors associated with recurrent stroke in Sikhio District, Nakhon Ratchasima Province, Thailand

**DOI:** 10.21542/gcsp.2024.43

**Published:** 2024-11-01

**Authors:** Atthawit Singsalasang, Jitra Bandidphak

**Affiliations:** Faculty of Public Health, Nakhon Ratchasima Rajabhat University, Thailand

## Abstract

**Background** Stroke survivors can experience residual disability and are at risk for subsequent strokes that might cause further damage or even death. The objective of this study was to identify factors associated with recurrent stroke in Sikhio District, Nakhon Ratchasima Province, Thailand.

**Method**: We used gender and age characteristics to match our study participants in a ratio of 1 case patient to 2 controls. The total sample size was 111 participants, 37 participants with recurrent stroke were considered as the case group and 74 participants without recurrent stroke were the control group. The case group was diagnosed by a physician along with a computerized tomography (CT) scan and received treatment at Sikhio Hospital in the period of 1 October 2021–30 September 2022. Face-to-face data were collected by a structured questionnaire and compared to the medical record.

**Results**: The study revealed that risk factors associated with recurrent stroke in Sikhio District were comorbidities (AOR = 4.64, 95% CI = 1.35–15.86, *p* < 0.014) and systolic blood pressure (AOR = 2.41, 95% CI = 1.10–5.78, *p* < 0.049).

**Conclusion**: Comorbidities and systolic blood pressure represented a risk to recurrent stroke among post-stroke patients. Therefore, hospitals should find effective methods to care for patients with co-morbidities and promote knowledge about blood pressure control.

## Introduction

Cerebrovascular disease (stroke) or paralysis/paresis is a common neurological condition, categorized into 2 types: ischemic stroke, which accounts for approximately 70–75% of cases, and hemorrhagic stroke, which represents around 25–30%. Although hemorrhagic stroke is less common than ischemic stroke, it tends to be more severe and is a major contributor to rising rates of disability and mortality. This presents a significant public health issue both in Thailand and globally^1^.

According to the World Stroke Organization (WSO)^2^, stroke is the second leading cause of death in worldwide, after ischemic heart disease. Globally, there are up to 12 million new stroke cases per year resulting in 6.5 million deaths. In addition, 89% percent of stroke-related death and disabilities occur in low and middle-income countries, with Asia having the highest number of stroke-related deaths and disabilities.

In Thailand, stroke is the second leading cause of death after cancer, and the third leading cause of disability. The Ministry of Public Health reported that in 2020, a total of 34,545 individuals died from stroke (mortality rate 53 per 100,000 people), with 19,949 males (58%), and 14,596 females (42%). Of these, 23,817 (69%) were over 60 years old. The incidence of stroke has been increasing annually. Between 2017 and 2020, the number of stroke cases per 100,000 population rose from 479 to 645. Similarly, the stroke mortality rates per 100,000 population were 48, 47, 53, and 53, during this period^3^.

Despite advances in treatment, many stroke survivors continue to experience disabilities, as well as risks of complications and recurrent strokes. Recurrent strokes exacerbate disability and increase mortality, with at least 1-in-4 stroke survivors experiencing a recurrent stroke. The recurrence rate is 14% within the first year and increases to 25% by the fifth year^4^. Additionally, ischemic stroke patients face a 5% annual risk of recurrence, which can rise to 10% within the first year. The risk of death from stroke or ischemic heart disease is approximately 7% per year. The risk of recurrence is even higher after the onset of stroke symptoms. It has been found that 12% of patients experience recurrent ischemic stroke within the first 30 days of diagnosis and 10% within the first 90 days after a transient ischemic attack^5^.

Health Zone 9, which includes the provinces of Nakhon Ratchasima, Buriram, Chaiyaphum and Surin, had the highest stroke rate in the country in 2021, with 257.72 cases per 100,000 population, according to a report from the National Health Security Office. The zone also recorded the highest number of stroke-related deaths, with 907 deaths (9.05%). Data from the Nakhon Ratchasima Public Health Office illustrated that Nakhon Ratchasima Province had the highest number of stroke-related deaths in Health Zone 9 and this number continues to rise. The stroke mortality rates for the province were 8.5%, 8.64%, and 8.95% in 2020, 2021 and 2022, respectively^6^. In Sikhio District, Nakhon Ratchasima Province, there were 355, 470, and 326 stroke cases in 2020, 2021, and 2022, respectively, with stroke mortality rates of 2.54%, 1.85%, and 3.03%. The rates of recurrent stroke in Sikhio District were 17.18%, 16.59% and 20.55% for the same period and these rates are expected to continue rising^7^.

Given the increasing incidence of stroke and the recurrence of vascular diseases, which contribute to higher disability and mortality rates, the researchers are interested in studying the factors associated with recurrent strokes. The PRECEDE Framework by Green & Kreuter^8^ was applied to the epidemiological diagnostic process as a guideline for studying and analyzing health problems, disease occurrence, various factors contributing to illness. This study aimed to investigate the factors associated with recurrent stroke in Sikhio District, Nakhon Ratchasima Province. The findings from this study can serve as a basis for policy creation, health care planning and development of a service system to help patients manage their health, prevent recurrent strokes, reduce morbidity and mortality from the cerebrovascular disease and maintain good health.

## Methodology

### Study design and population

This case-control study was matched using gender and age variables, with a case to control ratio of 1:2. The study population consisted of stroke patients who received treatment at Sikhio Hospital, Nakhon Ratchasima Province from October 1, 2021 to September 30, 2022, totaling 326 cases^7^. The sample group comprised patients with recurrent stroke and those without recurrence, all of whom had received services at Sikhio Hospital, Nakhon Ratchasima Province. The case-control sample size was calculated based on a literature review of risk factors for recurrent stroke of Sujittra, Weena & Plenpit^4^ using the Schlesselman^9^ formula as follows:

\begin{eqnarray*}n= \frac{[{{Z}_{\mathrm{\propto }}}_{\mathrm{/2}}\sqrt{2\overline{p}\overline{q}}+{Z}_{\mathrm{\beta }}\sqrt{{p}_{1}{q}_{1}+{p}_{0}{q}_{0}}]^{2}}{({p}_{1}-{q}_{0})^{2}} \end{eqnarray*}

The total sample size was 111 participants, divided into 37 participants with recurrent stroke in the case group and 74 participants without recurrence in the control group. All participants were diagnosed by a physician and underwent a computerized tomography scan confirming stroke.

The cases and controls were matched on two primary criteria: **gender** and **age** (±5 years). This matching process ensured that the cases and controls were comparable regarding these important demographic factors, thereby reducing potential confounding influences related to age and sex.

### Definition and assessment of comorbidities

Comorbidities were defined as the presence of any chronic medical conditions diagnosed prior to the initial stroke event, as documented in the patients’ medical records. These conditions included hypertension, diabetes, dyslipidemia, coronary artery disease, atrial fibrillation, and others commonly associated with increased stroke risk. The comorbidity status of each participant was assessed through a comprehensive review of their medical history, including laboratory results, medication use, and physician diagnoses, as recorded in the hospital’s health information system. Each comorbidity was classified as present or absent based on established diagnostic criteria.

### Research tool and validation

The data collection tool was a case recode form (CRF) consisting of four parts.

Part 1: Individual factors: gender, age, occupation, body mass index (BMI), comorbidities, stroke severity, blood pressure, blood sugar, blood lipid levels, duration of illness.

Part 2: Behavioral factors: smoking and alcohol consumption.

Part 3: Environmental factors: Medical appointment adherence and medication usage.

Part 4: Doctor’s diagnosis: post-stroke or recurrent stroke diagnosis and the number of recurrent strokes.

Three experts examined the content validity of the research tool. The Index of Item Objective Congruence (IOC) was greater than 0.5 (IOC: 0.67–1.00).

### Data collection

The data collection process was as follows: (1) A letter requesting permission to collect data was submitted to Sikhio Hospital. (2) Researchers collected data from the hospital’s medical record. (3) The collected data was checked and coded accurately in preparation for data analysis.

### Handling of missing data

Missing data was addressed using a complete case analysis approach, where only participants with complete data across all relevant variables were included in the final analysis. Prior to this, a thorough assessment of the extent and patterns of missing data was conducted. If missing values were less than 5% for a specific variable, listwise deletion was applied. In instances where missing data exceeded this threshold, imputation techniques such as mean imputation for continuous variables (*e.g.*, blood pressure, BMI) and mode imputation for categorical variables (*e.g.*, smoking status, comorbidities) were considered, provided these variables were not pivotal to the analysis. Sensitivity analyses were also performed to assess the potential impact of missing data on the study results.

### Ethical considerations

This research was approved by the Nakhon Ratchasima Rajabhat University Ethics Committee for Human Research (Ref. No. HE-072-2023). The research objectives, expected benefits, and research procedures were explained to the study population.

### Statistical analysis

Descriptive statistics, including the mean, percentage, and standard deviation (S.D.), were used to analyze the individual, behavior and environment factors of the sample. Inferential statistics, such as binary and multivariate logistic regression analyses, were performed to identify the factors associated with recurrent stroke. Additionally, the adjusted odds ratio (OR) and 95% confidence interval (CI) at a significance level of 0.05 were used to interpret the results.

## Results

### Individual factors

#### Case group (37 participants)

Most participants were male (56.76%), with 37.84% aged between 50–59 years. A majority (51.35%) were employed, and 62.16% had a BMI above the normal range. Comorbidities were prevalent in 89.19% of participants, with 90.91% having hypertension, 51.51% hyperlipidemia, and 45.45% diabetes. Stroke severity, measured by the NIHSS (National Institute of Health Stroke Scale), was classified as slight (0-4 points) in 59.46% of cases. Most participants had normal systolic blood pressure (81.08%), normal blood sugar levels (62.16%), normal cholesterol level (78.38%), normal triglyceride level (72.97%), normal low-density lipoprotein (LDL) levels (78.38%), However, 83.78% had abnormal high-density lipoprotein (HDL) levels. In terms of stroke duration, 45.95% had a stroke duration of less than 1 year and 62.16% had experienced a recurrent stroke twice (Table 1).

**Table 1:** Baseline characteristics of cases and controls.

**Characteristics**	**Cases**		**Controls**	
	**Number (n)**	**Percentage (%)**	**Number (n)**	**Percentage (%)**
**Gender**				
Female	21	56.76	42	56.76
Male	16	43.24	32	43.24
**Age** ** (Year)**				
<60 Year	18	48.65	34	45.95
≥60 Year	19	51.35	40	54.05
**Occupation**				
No occupation	11	29.73	21	28.38
Farmer/Employee/Shopkeeper	23	62.16	47	63.51
Monk	3	8.11	6	8.11
**BMI** ** (** **kg** **/** **m** ^**2**^ **)**				
Lower more criteria (<18.5)	3	8.11	9	12.16
Normal (18.5–22.9)	11	29.73	31	41.89
Over more criteria (>23.0)	23	62.16	34	45.95
	(*B* = 24.57, S.D. = 4.26, Min = 15.67, Max = 31.53)		(*B* = 23.75, S.D. = 4.96, Min = 15.91, Max = 42.57)	
				
**Comorbidities**				
No	4	10.81	21	28.38
Yes	33	89.19	53	71.62
**Severity of stroke level** ** (** **NIHSS)**				
Low	22	59.46	52	70.27
Middle-High	15	48.54	22	29.73
**Systolic blood pressure level** ** (** **mmHg** **)**				
<140	19	51.35	47	63.51
≥140	18	48.65	27	36.49
	(*B* = 146.92, S.D. = 18.935, Min = 119, Max = 187)		(*B* = 148.07, S.D. = 22.542, Min = 100, Max = 220)	
**Diastolic blood pressure level (mmHg)**				
<90	30	81.08	63	85.14
≥90	7	18.92	11	14.86
	(*B* = 82.70, S.D. = 10.888, Min = 65, Max = 111)		(*B* = 80.49, S.D. = 14.093, Min = 20, Max = 120)	
**Blood sugar levels** ** (** **mg** **/** **dl** **)**				
Normal (≤125)	23	62.16	48	64.86
High (>125)	14	37.84	26	35.14
	(*B* = 118.59, S.D. = 42.908, Min = 60, Max = 289)		(*B* = 123.99, S.D. = 55.013, Min = 56, Max = 299)	
**Cholesterol** ** (mg/dl)**				
Normal (≤200)	29	78.38	54	72.97
Abnormal (>200)	8	21.62	20	27.03
	(*B* = 163.14, S.D. = 54.968, Min = 67, Max = 346)		(*B* = 175.86, S.D. = 48.020, Min = 52, Max = 293)	
**Triglyceride** ** (mg/dl)**				
Normal (≤150)	27	72.97	55	74.32
Abnormal (>150)	10	27.03	19	25.68
	(⋅ = 129.22, S.D. = 68.490, Min = 51, Max = 315)		(⋅ = 129.65, S.D. = 72.905, Min = 25, Max = 381)	
**HDL** ** (mg/dl)**				
Normal (≥60)	6	16.22	13	17.57
Abnormal (<60)	31	83.78	61	82.43
	(*B* = 47.73, S.D. =, 13.702, Min = 26, Max = 78)		(*B* = 47.36, S.D. = 15.467, Min = 22, Max = 117)	
**LDL** ** (** **mg** **/** **dl** **)**				
Normal (≤130)	29	78.38	51	68.91
Abnormal (>130)	8	21.62	23	31.08
	(*B* = 95.65, S.D. = 49.349, Min = 18, Max = 263)		(*B* = 105.34, S.D. = 41.913, Min = 21, Max = 222)	
**Smoking**				
No	19	51.35	39	52.70
Yes/Have been smoking	18	48.65	35	47.30
**Alcohol drinking**				
No	15	40.54	34	45.95
Yes/Have been drinking	22	59.46	40	54.05
**Appointment** ** for medical treatment**				
Every time by appointments	17	45.95	51	68.92
Sometimes/not on time by appointments	20	54.05	23	31.08
**Medicine use**				
Not miss medicine	16	43.24	49	66.22
Missed medicine of their congenital disease/stopped their own medicine	21	56.76	25	37.78

#### Control group (74 participants)

Most participants were male (56.76%) with 35.14% aged between 50–59 years, A majority (52.70%) were employed, and 45.95% had a BMI above the normal range. Comorbidities were present in 71.62% of participants, with 83.01% having hypertension, 50.94% diabetes, and 37.74% hyperlipidemia. Stroke severity was classified as slight (0-4 points) in 70.27% of participants. Most participants had normal systolic blood pressure (63.51%), normal blood sugar levels (64.86%), normal cholesterol levels (72.97%), normal triglyceride levels (74.32%), normal low-density lipoprotein (LDL) levels (68.91%). However, 82.43% had abnormal HDL levels.

### Behavioral factors

#### Case group (37 participants)

Most participants did not smoke (51.35%), 32.43% had a history of smoking but had quit and 16.22% were current smoker. Regarding alcohol consumption, 51.35% had previously consumed alcohol but had stopped, 40.54% did not drink, and 8.10% were still drinking.

#### Control group (74 participants)

Most participants did not smoke (52.70%), 28.38% were current smoker and 18.92% had a history of smoking but had quit. In terms of alcohol consumption, 45.95% did not drink, 32.43% had previously consumed alcohol but had stopped, and 21.62% were still drinking.

### Environment factors

#### Case group (37 participants)

Most participants sometimes missed or were late for medical appointments (54.05%) while 45.95% attended all their appointments. Additionally, 56.76% missed doses or stopped taking their prescribed medication for comorbid conditions, while 43.24% adhered to their medication regimen.

#### Control group (74 participants)

Most participants attended all their medical appointment (68.92%), while 31.08% sometimes missed or were late for appointments. Moreover, 66.22% adhered to their prescribed medication for comorbid conditions, while 33.78%, missed doses or stopped taking their medications (see Table 3).

### Factors associated with recurrent stroke

Bivariate analysis indicated that comorbidities, stroke severity (NIHSS), systolic blood pressure, medical appointment adherence, and medication adherence were significantly associated with recurrent stroke (*p*-value < 0.25) among post-stroke patients treated at Sikhio hospital, (see Table 2).

**Table 2:** Factors associated with recurrent stroke in Sikhio District, Nakhon Ratchasima Province. Crude analysis (*n* = 111).

**Factors**	**Case** **n (%)**	**Control** **n (%)**	**Crude OR**	**95% CI** **of OR**	**p-value**
**Gender**					1.00
Female	16 (43.24)	32 (43.24)	1		
Male	21 (51.76)	42 (51.76)	1.00	0.45–2.22	
**Age** ** (** **Year** **)**					0.788
<60 Year	18 (48.65)	34 (45.95)	1		
≥60 Year	19 (51.53)	40 (54.05)	0.89	0.41–1.97	
**Occupation**					
No occupation	11 (29.73)	21 (28.38)	1		0.988
Farmer/Employee/ Shopkeeper	23 (62.16)	47 (63.51)	0.93	0.38–2.26	
Monk	3 (8.11)	6 (8.11)	0.95	0.19–4.57	
**BMI** ** (** **kg** **/** **m** ^**2**^ **)**					0.269
Lower more criteria (<18.5)	3 (8.11)	9 (12.16)	1		
Normal (18.5-22.9)	11 (29.739)	31 (41.89)	1.06	0.24–4.66	
Over more criteria (>23.0)	23 (62.16)	34 (45.95)	2.02	0.49–8.31	
**Comorbidities**					0.028
No	4 (10.81)	21 (28.38)	1		
Yes	33 (89.19)	53 (71.62)	3.26	1.03–10.36	
**Severity of stroke level** ** (** **NIHSS)**					0.258
Low	22 (59.46)	52 (70.27)	1		
Middle-High	15 (40.54)	22 (29.73)	1.61	0.70–3.67	
**Systolic blood pressure level** ** (** **mmHg** **)**					0.220
<140	19 (51.35)	47 (63.51)	1		
≥140	18 (48.65)	27 (36.49)	1.64	0.74–3.66	
**Diastolic blood pressure level (mmHg)**					0.588
<90	30 (81.08)	63 (85.13)	1		
≥90	7 (18.92)	11 (14.87)	1.33	0.47–3.79	
**Blood sugar levels** ** (** **mg** **/** **dl** **)**					0.780
Normal (≤125)	23 (62.16)	48 (64.86)	1		
High (>125)	14 (37.84)	26 (35.14)	1.12	0.49–2.54	
**Cholesterol** ** (mg/dl)**					0.532
Normal (≤200)	29 (78.38)	54 (72.97)	1		
Abnormal (>200)	8 (21.62)	20 (27.03)	0.74	0.29–1.90	
**Triglyceride** ** (mg/dl)**					0.878
Normal (≤150)	27 (72.97)	55 (74.33)	1		
Abnormal (>150)	10 (27.03)	19 (25.67)	1.07	0.43–2.62	
**HDL** ** (mg/dl)**					0.858
Normal (≥60)	6 (16.22)	13 (17.57)	1		
Abnormal (<60)	31 (83.78)	61 (82.43)	1.10	0.38–3.17	
**LDL** ** (** **mg** **/** **dl** **)**					0.288
Normal (≤130)	29 (78.38)	51 (68.92)	1		
Abnormal (>130)	8 (21.62)	23 (31.08)	0.61	0.24–1.54	
**Smoking**					0.893
No	19 (51.35)	39 (52.70)	1		
Yes/Have been smoking	18 (48.65)	35 (47.30)	1.05	0.47–2.32	
**Alcohol drinking**					0.588
No	15 (40.54)	34 (45.95)	1		
Yes/Have been drinking	22 (59.46)	40 (54.05)	1.24	0.56–2.77	
**Appointment** ** for medical treatment**					0.038
Every time by appointments	17 (45.95)	51 (68.92)	1	1.16–5.88	
Sometimes/not on time by appointments	20 (54.05)	23 (31.08)	2.61		
**Medicine use**					0.037
Not miss medicine	16 (43.24)	49 (66.22)	1	1.15–5.78	
Missed medicine of their congenital disease/stopped their own medicine	21 (56.76)	25 (33.78)	2.57		

**Table 3:** Factors associated with recurrent stroke in Sikhio District, Nakhon Ratchasima Province. Multivariate analysis (*n* = 111).

**Factors**	**Case** **n (%)**	**Control** **n (%)**	**Crude OR**	**Adjusted** **OR (AOR)**	**95% CI** **of AOR**	**p-value**
**Comorbidities**						0.014
No	4 (10.81)	21 (28.38)	1	1		
Yes	33 (89.19)	53 (71.62)	3.26	4.64	1.35–15.86	
**Severity of stroke level** ** (** **NIHSS)**						0.145
Low	22 (59.46)	52 (70.27)	1	1		
Middle-High	15 (40.54)	22 (29.73)	1.61	1.92	0.80–4.61	
**Systolic blood pressure level** ** (** **mmHg** **)**						0.048
<140	19 (51.35)	47 (63.51)	1	1		
≥140	18 (48.65)	27 (36.49)	1.64	2.41	1.10–5.78	
**Appointment** ** for medical treatment**						0.128
Every time for appointments	17 (45.95)	51 (68.92)	1	1		
Sometimes/not on time for appointments	20 (54.05)	23 (31.08)	2.61	2.04	0.81–5.11	
**Medicine use**						0.897
Not miss medicine	16 (43.24)	49 (66.22)	1	1		
Missed medicine of their congenital disease/stopped their own medicine	21 (56.76)	25 (33.78)	2.57	0.85	0.67–10.68	

Multivariate analysis, using multiple logistic regression, further identified these factors as significant predictors of recurrent stroke. Specifically, participants with comorbidities had a 4.64 times higher risk of recurrent stroke (AOR = 4.64; 95% CI [1.35–15.86]; *p*-value = 0.014) compared to those without comorbidities. Participants with systolic blood pressure ≥ 140 mmHg had a 2.41 times higher risk of recurrent stroke (AOR = 2.41; 95% CI [1.10–5.78]; *p*-value = 0.048) compared to those with systolic blood pressure <140 mmHg (see Table 3).

## Discussion

In Sikhio District, two statistically significant factors were identified as being associated with recurrent stroke: comorbidities and systolic blood pressure. Stroke patients with comorbidities were 4.64 times more likely to experience recurrent strokes compared to those without comorbidities (AOR = 4.64, 95% CI = 1.35–15.86, *p* = 0.014). The comorbidities among the sample included hypertension (90.91%), hyperlipidemia (51.51%), and diabetes (45.45%). According to the study of Piyanan Temprom et al. ^10^ which examined the prevalence of high recurrence risk and associated factors in patients with acute cerebral ischemia, comorbidities were found to be significantly associated with a high risk of recurrence. Hyperlipidemia (AOR = 3.14, 95% CI = 2.64−3.74) and atrial fibrillation (AOR = 1.82, 95% CI = 1.21−2.72) were particularly notable.

Diabetes is another significant comorbidity that contributes to endothelial damage due to hyperglycemia and the glycation process. As glycation progresses, pancreatic beta cells responsible for insulin production diminish, resulting in abnormalities in blood adipokines.

Furthermore, dyslipidemia was found to increase the risk of ischemic stroke by nearly three times, largely due to the involvement of low-density lipoprotein (LDL) in the formation of arteriosclerotic plaque. Poor control of blood lipid levels accounts for 20% of recurrent strokes, increasing the risk by 1.5 times. Patients taking statins were shown to reduce stroke risk by 29%, and LDL levels may be associated with a history of diabetes^11^.

According to Lambert^12^, diabetes causes 9% of recurrent strokes and increases the risk of recurrence threefold. High blood sugar thickens blood vessel walls, leading to impaired circulation and insufficient blood supply to the brain^13, 14^.

Stroke patients with systolic blood pressure ≥140 mmHg had a 2.41 times higher risk of recurrent stroke compared to those with systolic blood pressure <140 mmHg (AOR = 2.41, 95% CI = 1.10−5.78, *p* = 0.048). According to Somsak Thiamkao^12^, poor blood pressure control was the primary cause of recurrent stroke (33.75%). A study by Wilaiporn Puttawong^15^ in Phayao Province also found that systolic blood pressure >160 mmHg was significantly associated with stroke risk (OR = 237.1, 95% CI = 86.2–652.2). This finding aligns with the study by Jutathip Thepsuwan^16^ which revealed that high blood pressure ≥ 140/90 mmHg increases the risk of recurrent stroke by 3–17 times.

High blood pressure thickens and hardens blood vessels, leading to arterial stenosis and cerebral ischemia, eventually damaging brain tissue and impairing bodily functions. A further study^17^ also found that diastolic blood pressure is a contributing factor to recurrent strokes in middle-aged and young patients in China. Failure to control blood pressure below 140/90 mmHg can lead to arterial wall thickening, arterial stenosis, and cerebral ischemia, ultimately leading to brain tissue damage. Thus, high blood pressure significantly increases the likelihood of stroke, while proper blood pressure control reduces the risk of recurrence.

### Comparisons with previous studies

Our findings on stroke recurrence are in line with previous research conducted in Thailand and Southeast Asia. For example, the increased recurrence risk among patients with comorbidities like hypertension and diabetes mirrors earlier findings^10^, where a significant association between hyperlipidemia and stroke recurrence was reported. Additional researc *h*^13^ further supports our conclusion that poor blood pressure control significantly increases the risk of stroke recurrence.

Comparisons to studies from Southeast Asia are particularly relevant due to shared lifestyle factors, healthcare access, and genetic predispositions across the region. Puttawong’s^15^ study in Phayao Province, for instance, found that systolic blood pressure >160 mmHg greatly increases stroke risk, echoing our results, which showed that systolic blood pressure ≥140 mmHg increases the recurrence risk by 2.41 times. These studies collectively highlight the importance of managing blood pressure and comorbid conditions such as diabetes and dyslipidemia to prevent recurrent strokes.

### Impact of unmeasured confounders

While our study adjusted for key factors such as age, gender, and comorbidities, there remains the possibility of unmeasured confounders influencing the results. Variables such as socioeconomic status, which affects access to healthcare and medication, adherence to treatment beyond medication usage, and detailed dietary factors were not assessed. These factors may significantly impact stroke recurrence by influencing patients’ ability to manage key risk factors like blood pressure and cholesterol levels. Moreover, lifestyle habits such as physical activity, smoking, and alcohol consumption, which were not captured in this study, could also play a role in stroke recurrence. Future research should aim to include these unmeasured variables to provide a more comprehensive analysis of the factors contributing to stroke recurrence. Doing so will allow for more targeted interventions, particularly in addressing socioeconomic barriers to effective disease management and promoting adherence to long-term treatment plans.

## Conclusion

Two significant factors; comorbidities and systolic blood pressure were found to be associated with recurrent strokes. Public health services should prioritize preventive measures, monitoring, evaluation, and screening of high-risk populations and stroke patients. Stroke patients with comorbidities such as hypertension, diabetes, and dyslipidemia should be closely monitored and assessed to reduce the likelihood of recurrent strokes.

## Limitations

This study is subject to potential selection bias, as participants were selected based on hospital records, which may not represent the general population of stroke patients in the region. The use of hospital-based controls might skew the findings toward individuals with better access to healthcare. In addition, due to the retrospective nature of the study, there is a risk of recall bias, especially when participants were asked about behavioral factors such as smoking or alcohol use. Stroke survivors might underreport or misremember certain behaviors.

## Acknowledgement

The author thanks to the research data from Sikhio hospital, Nakhon Ratchasima Province, Thailand, for this study.

## Competing interest

The authors declare that they have no competing interest.
